# Development and psychometric properties of an instrument to measure perception of aphrodisiac use among undergraduates in a southwestern Nigerian university

**DOI:** 10.1186/s12889-024-18736-y

**Published:** 2024-05-17

**Authors:** Olawumi Cecilia Fatade, Gabriel Ifeoluwa Makinde, Ayodeji Matthew Adebayo

**Affiliations:** Department of Community Medicine, College of Medicine, University of Ibadan, Ibadan, Nigeria

**Keywords:** Aphrodisiacs, Psychometric analysis, Polychoric analysis, University undergraduates

## Abstract

A tool to measure perception of aphrodisiac use by undergraduates students of University of Ibadan, Oyo State, Nigeria was developed and validated. The study was hinged on several theories that could explain potential to use aphrodisiac among the youths. An exploratory mixed methods design was used to develop a scale to measure perception of aphrodisiac use by undergraduate students of University of Ibadan. Qualitative data collection was performed among thirty equally represented male and female students and five key informant interview participants while 919 participants completed the quantitative phase (surveys). Integration of matched qualitative themes from FGD/KII to survey domains was achieved through the ‘building approach’. Qualitative themes assessing perceptions of aphrodisiac use by university undergraduate students were used to develop original survey items as well as new survey items peculiar to research subjects. Exploratory factor analysis was deployed on polychoric correlation matrix of the items using R-statistical packages. Further model fit analysis was conducted using confirmatory factor analysis on the items suggested by EFA as well as composite reliability and construct validity tests for the constructs. Mean Z-scores of factors were computed against socio-demographics and symptoms of aphrodisiac use among respondents that have ever used it. Most respondents (84.3%) were under 25 years, mostly male (58.4%) and singles (96.3%), with 41.3% earning ≤20,000 naira monthly. The enhanced content validity of the items from mixed method analysis yielded two major domains. Two succession of factor analyses and a structural equation modeling suggested that a first-order model is good fit for experimental data (TLI = 0.931; CFI = 0.948; SRMR = 0.047; RMSEA = 0.083). The four-factor solution to the model included: prolonged sexual performance, use without erectile dysfunction or medical advice, treatment of erectile dysfunction and recreational purposes with an internal and composite reliability that ranged from 0.62–0.92 and 0.63–0.92. The validation with socio-demographics and consequences of aphrodisiac use indicated that: Male respondents, those older than 20years, the married, those from poorly educated parent and sufferers of all related consequences had statistically significant differences with poor perception of aphrodisiac use’ domains. This validated instrument is good for assessment of perception of aphrodisiac use among students in tertiary institution albeit with caution. A version of the scale that is broadened with highly refined items and tested for high internal validity is suggested.

## Background

Aphrodisiacs are substances that increase sexual desire [1]. They can also be defined as any foods or drugs that arouse sexual instinct, induce venereal desire, and increase pleasure and performance [2]. There are certain animal and plants-based drugs, foods and drinks substances including certain human behaviours which have reputation for making sex more attainable and pleasurable [3]. However, few of these were subjected to pharmacological processes which include clinical trials protocols for approval or recognized in significant research [4, 5].

The main indication for use of aphrodisiac drugs is erectile dysfunction [6] however, the qualities and benefits of sexual pleasures of enhanced libido and erection have prompted their indiscriminate and excessive use [7]. A study reported that most of the users are between 15 and 30 years old (Makwana et al., 2013). There is a rising demand and use of aphrodisiacs among young people without any medical indications [8–10]. Young people have high desire to explore and increase sexual performance and anecdotal evidence has shown that males are more vulnerable to using recreational aphrodisiacs [11].

Improper use of aphrodisiac drugs can bring potentially often preventable cause of health hazards that range from disease conditions to death more so evidences are mounting about commonness of adverse reactions to medicines in recent times. Abuse of aphrodisiacs for recreational purpose by young adults with or without knowledge of its debilitating health implications are often done through self-medication (SM) [7]. Self-medication, which is the selection and use of medicines by individuals to treat self-recognized illnesses or symptoms without consultation of health care professional, was reported in a study among Ghanian men [12]. Majority were reported to be using sex-enhancing medications without any medical reason [12]. This act was further substantiated with the fact that about 75% Ghanaians men hold the conviction that intravaginal ejaculatory latency of 7–25 min is adequate as against the 3–7 min sex therapist recommended [12].

For young adults, the dependency on aphrodisiac for elongating sexual pleasures may have started out of curiosity and experimentation however, it ultimately advance to addiction and other serious risky behaviours such as substance use and engagement in multiple sexual relationships [13]. According to a survey of US college students, lifetime and past year prevalence of nonmedical prescription benzodiazepine use was 8% and 5%, respectively. Risky factors found among the students included higher rates of substance use and bisexual activities [14]. In Nigeria, the use of drugs for non-medical purposes by youth is not uncommon, however the rate at which these leaders of tomorrow embrace these drugs for various reasons has been described as alarming [15].

Young people who persistently abuse aphrodisiacs and other substances are liable to experience array of problems, including academic difficulties, health-related problems (including mental health and sexual and reproductive), poor peer relationships, and involvement with the juvenile justice system [16, 17]. These health challenges are definitely with huge burden of concern to an already saturated public health system in Sub-Saharan Africa [18] [7].

There is a dearth of studies on perception of aphrodisiac use among men. However, a qualitative study among Ghanian men gave indication that combined involvement of a complex interactions between social, psychological and biological factors influence use of aphrodisiac [19]. The findings on social factor necessitating aphrodisiac use were based on men’s perception that sexual ability is a function of status and prestige in the society [19]. The psychological and biological reasons for the use of aphrodisiacs were to punish women who materially and financially exploit men and for proving masculinity in bed activities during advancing ages and disease conditions [19]. The disadvantage of this study was that quantitative measurements to test the external generalizability of the study findings on socio-demographic and symptomatic characteristics was not conducted.

Studies that endeavor to understand perceptive rationale for use of aphrodisiac among young adults are rare in Nigeria. This is because of lack of culturally/locally grounded tools to measure the constructs.

Thus, this study was aimed at determining the psychometric properties of an instrument developed to assess the perception to use of aphrodisiacs among tertiary education students in University of Ibadan. Findings derived from this study can assure of precision and trust in the outcome of evaluation should such a tool be applied in future similar studies.

## Methodology

### Study design

The study employed an exploratory mixed methods design, where the qualitative phase of data collection and analysis preceded the quantitative phase of data collection and analysis [20]. The design was applied to develop an instrument to measure perception of aphrodisiac use among undergraduate youths in a Nigerian university.

### Description of the study area

The study was carried out in the University of Ibadan, Ibadan, Oyo State, Nigeria. The University of Ibadan, situated in Ibadan North Local Government Area is the oldest Nigerian university established in 1948 as an external College of University of London. It’s thirteen faculties currently holds about 35,000 students. The university’s undergraduate halls of residence accommodate over 8,000 female and male students.

### Study population

For the qualitative survey, pharmacists and drug peddlers within Ibadan North LGA constituted key informants interview participants while undergraduates in the University of Ibadan comprised the respondents of the Focus Group Discussion in the qualitative aspect of the data collection. The quantitative component of the study consisted of male and female undergraduate students who were aged between16 years and above enrolled into courses at the University of Ibadan. Eligibility criteria for participation was being a male or female undergraduate student of the University of Ibadan and being aged 16 years and above while those who were not students of the university and those below sixteen years of age were excluded from the study.

### Qualitative data collection and analysis

#### Focus group discussion and key informant interview (composition and conduct)

Four sessions (two each in male and female residential halls) of tape recorded FGD were conducted with the permission of the thirty participants whose identities were concealed by denoting them with letter R. Each discussion lasted for 40–50 min. An open-ended interview protocol was used to guide the discussion. The FGD sessions were facilitated by a trained facilitator while two research assistants participated as note taker and observer. More FGD sessions were not conducted because data saturation was reached.

The KII sessions were conducted in English language and each participant’s consent was obtained verbally. Five KII sessions were conducted in Ibadan North LGA. The KII consisted of five key informants; three pharmacists and two drug peddlers. The key infomants were purposively selected. The anonymity of participants in the KII sessions was also protected in the report. An open-ended interview protocol was used to guide the discussion by a trained facilitator.

The audio-recorded FGD sessions were transcribed and analyzed by an independent expert in qualitative methods. Deductive content analysis was conducted on the transcripts to identify and categorize the resulting themes into perception of aphrodisiac use domains. Analysis proceeded until data saturation when no new dimensions were identified in the data.

#### Mixed method data integration (building approach)

In this exploratory sequential mixed methods design, we used the building approach for our method-level data integration, where the themes on perception of use of aphrodisiac and participant’s quotes from the initial qualitative phase were used to adapt existing survey items of survey (quantitative phase) and development of new survey items and. The existing survey items developed from literature review and FGD were re-written to allow for cultural adaptation for the intended population. Fifteen survey items, including new and adapted items were compiled using this approach. These items were assessed for face and content validity by experts in community medicine including sub-specialties like reproductive and family health, and clinical epidemiology. Other items on the questionnaire are socio-demographic characteristics and psychological and health items perceived to be linked to use of aphrodisiac. From this list, those that prompt poor perception of aphrodisiac use were rightly assigned likert score that ranged from 1 to 5 on a strongly agree to strongly disagree scale. While items that favour good perception were reversely coded. This was aimed at ensuring scores from each domains of aphrodisiac perception use and total scores reflected final categorization of good and poor perception.

#### Reporting of mixed methods data integration (merging approach)

Finally, a joint presentation of integrated data (merging approach) occurred at the reporting level to create a joint display of qualitative and quantitative data. The initial process required is matching the qualitative themes to their corresponding perception of aphrodisiac use survey item domains because of the mainly deductive approach used to conduct the content analysis of the focus group transcripts. Under each relevant theme, sample quotes that had been used to create the specific culturally adapted items were added to the joint display, along with their corresponding items, showing the integration of the qualitative phase with the quantitative phase. After a preliminary quantitative analysis, mean item scores and item-total correlations were added to the final joint display to evaluate congruence between the two phases.

### Sample determination and sampling technique for quantitative aspect

The minimum sample size of 410 male and female students of University of Ibadan was estimated using Leslie Kish sample size formula for determining single proportion for descriptive studies. Multistage sampling technique involving the use of simple random technique by balloting was deplored for data collection at each required stage of recruitment protocol. At stage one, a total of four halls comprising of two male halls out six and two out of three females’ halls of residence were selected. Five blocks were selected from the halls of interest making a total of 20 blocks in the second phase of the multistage sampling process. At least 20 rooms were selected from each of the selected blocks at the third stage of recruitment processes. Finally, at least one respondent was recruited from each selected room that comprised the blocks of interest.

## Data analysis

### Factor reduction analysis

Polychoric correlation, used in analyzing ordinal and nominal likert scale, was used to elicit the matrix needed for factor analysis of the original fifteen items [21]. The assumption behind polychoric correlation coefficient is that pairs of ordinal scores are generated by latent bivariate normally distributed random variables. Measuring the association between ordinal variables entails the estimation of the product moment correlation between the corresponding normally distributed variables. The polychoric correlation matrix was estimated using the two-stage procedure described in Lee et al. [22] and implemented in R statistical packages [21]. Note that since the matrix was estimated in a pairwise fashion, it was possible to be non-positive definite. Furthermore, an exploratory factor analysis (EFA) was then performed on the estimated polychoric correlation in R.

Confirmatory factor analysis (CFA) was performed from the latent variables obtained from EFA. Here the latent items of the scales must prove their relatedness with their respective unobserved constructs according to acceptable goodness of fit measures. The measurement parameters are: Comparative Fit Index (CFI); Tucker Lewis Index (TLI); Standardized Root Mean Square Residual (SRMR) and Root Mean Square Error of Approximation (RMSEA). CFI analyses the change in fit between the hypothesized model and the multidimensional model [23, 24] which ranges between 0 and 1. TLI indicates the total co-variation in the model and ranges between 0 and 1. The values of TLI and CFI greater than 0.90 imply a good fit to the data [23, 24]. RMSEA is based on analysis of residuals and its expected value for a good model data fit is one with less than 0.08 [23, 24]. The value of (RMSEA) shows sensitivity to degree of freedom and complexity of the proposed model (Kline et al., 2011; Wang, 2012) [23, 24]. SRMR is an index of the average of standardized residuals between the observed and the hypothesized covariance. It indicates a good fit when it produces a value less than 0.05 [23].

### Internal reliability

Internal consistency reliability measures the extent to which all items within a scale are indeed capturing the same construct. Cronbach’s alpha coefficients greater than 0.80 indicate high levels of internal consistency, while values that range between 0.70 and 0.61 suggest acceptable internal consistencies [25]. This was tested in SPSS statistical packages IBM version 20.0.

Further reliability methods of composite reliability and construct validity of convergent/divergent analyses were conducted on the determined constructs to strengthen their validity. The composite reliability was analysed using an online calculator [26] while convergent/divergent were performed with SPSS statistical packages IBM version 20.0.

### External validity

For external generalizability validation of the items, data on socio-demographic characteristics, medical and psychological impact of use of aphrodisiac by respondents were listed in the questionnaire.

Information of a proforma on sociodemographic characteristics of university students was adapted and incorporated into the data collection instruments.

Psychological symptoms of sex addiction, loss of sex performance self-esteem and sexual perversion deduced from focus group discussion and KII were developed for respondents to self-report their certainty that the symptoms they experienced were caused by their dependency on aphrodisiac substances through a yes or no response.

Characteristics of physical symptoms of priapism and fatigue adapted from focus group discussion and KII were developed by asking respondents to self-report their confidence that their use of aphrodisiac substances for sexual performance was responsible for the occurrence of the symptoms in a yes or no response.

## Results

### Pretest report

Forty-two questionnaires were pre-tested among undergraduates at Lead City University, Ibadan, which were not part of the study population but were similar to the main study subjects by characteristics and socio-demography. The pre-test excluded respondents who were not present on campus as at the time of collecting data. The outcome of the pretest was used to revise ambiguous questions and adding of information found useful for improving the quality of the questionnaire.

### Respondents’ socio-demographic characteristics

Majority (84.3%) of the respondents were less than 25 years of age, 58.4% were males, 96.3% singles and 41.3% earned less than or equal 20,000 naira as average monthly income. The largest percentage of the subjects where from Yoruba ethnic background (73.6%) and more than one-half of either of the respondents’ parents had tertiary education (father = 59.8%; mother = 54.2%) Table 1.

**Table 1:** Socio-demographic characteristics of respondents

Characteristics	*n*	%
Age group (years)		
16–24	775	84.3
25–40	144	15.7
**Gender**		
Male	537	58.4
Female	382	41.6
**Marital status**		
Single	885	96.3
Married	34	3.7
**Ethnicity**		
Yoruba	676	73.6
Ibo	72	7.8
Hausa	117	12.7
Others	39	4.2
Missing	15	1.6
**Father’s educational level**		
No formal education	100	10.9
Primary	64	7.0
Secondary	156	17.0
Tertiary	550	59.8
Missing	49	5.3
**Mother’s educational level**		
No formal education	96	10.4
Primary	90	9.8
Secondary	175	19.0
Tertiary	498	54.2
Missing	60	6.5
**Average monthly income (Naira)**		
≤ 20,000	380	41.3
> 20,000	121	13.2
Missing	418	45.5

### Qualitative result

Three questions that themed on perceived indication for use of aphrodisiac by the youth, perceived benefits of using aphrodisiacs and perceived side effects of using aphrodisiacs were discussed among FGD interviewees and KII.

What are your perceived indication for use of aphrodisiac by the youth?

Respondents’ perceived use of aphrodisiac substances include derivation of prolonged sexual pleasure.

Aphrodisiacs are basically used for sexual pleasure as narrated by one of the participants: *‘You know to gain libido and readiness for sexual action sometimes, students even improvised and go to extent of inventing a concoction of soaked cassava flakes (Garri) and paracetamol. For example, someone told me that one can take ‘Garri’ and put Paracetamol inside it would serve the same purpose of making you sexually active and ready for sexual action. ’< R6_FGD_Male* Undergrad_UI_ Hall 2>.

*Another said ‘I think it is that mixing that people use the most: like, they mix with drinks and all of that’.< R4_FGD_Male* Undergrad_UI_ Hall 2>.

Opinions on indication for aphrodisiac use were split among KII participants. One of them supported use of aphrodisiac for attainment of sexual pleasure.

*Yes there are indications for use by youths as well. Usually it is not on medical basis, it is just on leisure basis to improve sexually performance basically. Young or unmarried people uses basically for sexual pleasure to improve performance to impress their partners’*.*< KII* 2_Pharmacist_UCH_IBNLGA>.

Another group believed there are indications for aphrodisiacs use by young adults.

*‘*‘*There are indications for uses. Actually medically, they are some youths that really need it so it is medically indicated and recommended’< KII* 3_Pharmacist_UI_IBNLGA>.

Youths diagnosed with erectile dysfunction or who cannot perform sexually well should be free to use them while only one argued against the use among the subjects. ‘*Yes, why not. I can advise youth to buy and use it because if a young man has a small manhood, we do give them the man power drug. For those with ‘Atosi-Inu‘(internal reproductive system infection), we do give them gonorrhea drugs’*. *< KII* 4_Drug Peddlar_IBNELGA>.

Some others said there are no indications for the use of aphrodisiacs by youths no matter how it is argued; hence, it is an abuse:

‘*Usually not. There is no indication for aphrodisiac use among the youths. Let’s talk about the youth thing. You know sometimes they want to enhance performance without sex but we don’t have them as many as the middle aged maybe because vigor is still very much in youth…*’ *< KII* 1_Pharmacist_Moko_IBNLGA>.

What are the perceived benefits of using aphrodisiac?

Participants agreed to varieties of benefits associated with the use of aphrodisiac products interestingly prior to being prompted or probed. Some of the benefits they perceived are obtainable include enhancement and boosting of self-esteem during sexual intercourse, improved sexual performance through sustained and prolonged erection, vaginal tightening, sexual gratification and self-satisfaction.

What are the perceived side effects/implications of use of aphrodisiacs?

Series of implications were highlighted to be associated with the use of aphrodisiacs. To our discussants, the side effects include:

Psychological: participants listed psychological conditions of addiction to aphrodisiac substances, sexual perversion that could lead to pedophilia, low self-esteem, mental illness etc.

*‘Because they can get addicted to the usage and the addiction might lead to them having uncontrolled and contnous sexual urge leading them to have sex with anybody available including vulnerable persons like little children* *< R5_FDG_Female* Undergrad_UI_ Hall 4>, *< R6_FDG_Female* Undergrad_UI_ Hall 4>.

*‘Madness is associated with aphrodisiac’< R1_FGD_Female Undergrad_UI_ Hall 3> -*.

Medical: low blood pressure, headaches, unwanted erections, contaminations, disease infections, weakness etc-

**‘***Most of the side effects are low blood pressure, headaches, enhanced unwanted erections. Then on long term basis, erectile dysfunction. During sexual activity, there can be penile fracture. There has been reported cases of penile fracture’< KII* 2_Pharmacist_UCH_IBNLGA>.

‘*I would like to say that because it is locally made, it might not be safe, like it might be polluted in any form so that’s also part of this. There are more side effects in local ones compared with medical ones’< R5_FDG* Female Undergrad_UI_ Hall 4>.

It could cause fatigue due to abnormally prolonged sexual activity.

*< R3_FGD_Male* Undergrad_UI_ Hall 1>.

*It could cause alter natural sexual desire due excitatory disruptions caused by the aphrodisiac substances’.*

*< R4_FGD_Female* Undergrad_UI_ Hall 4>.

On the contrary, other FGD discussants did not accept these assertions: affirming none availability of harms in the use of aphrodisiacs. To them, *these substances are more profiting*, *doing good than harm*’. *Therefore, it is not as bad as people perceived they were*. As such, there are no associated harm. As they opined:

*The one I just mentioned are made of herbs, they are naturally made so their side effect is not negative or may not be as much orthodoxly produced ones. Its function is latent*.

*< R6_FGD* Male Undergrad_UI_ Hall 2>, *< R1_FDG_Female* Undergrad_UI_ Hall 4>.

Some KII participants also supported this assertion thus:

*‘No side effect because we used only herbs to prepare it…. but for others like man power, orthodox, there are many side effects. I advise men not to be using man power (orthodox medicine) because it has a long term effect on the heart and hormones of men. Also women that have sexual intercourse with men that uses man power, it affects the womb of women because it is a dangerous chemical*’ *< KII* 5_Drug Peddlar_IBNELGA>, *< KII* 1_Pharmacist_Moko_IBNLGA>.

Financial/Economical problems

‘*it would render you broke, it would stop your money, it would affect you financially’*. *< R8_FGD_Male* Undergrad_UI_ Hall 1>.

### Mixed method results

The joint display showing the perception of aphrodisiac use domains, qualitative themes and sample quotes, corresponding new culturally adapted items, mean item scores, and item-total correlations are presented in Table 2. Themes from the FGD which included ‘perceived indication for use of aphrodisiac’, ‘perceived benefits of using aphrodisiac’ and ‘perceived side effects/implications of use of aphrodisiacs’ were assessed against the constructs from the structural equation modelling of the scale. Two constructs of perception on use of aphrodisiac for prolonged sexual performance and recreational purpose matched the deductive theme on perceived benefits of aphrodisiac use. The theme on indication for aphrodisiac use matched two constructs of perception of aphrodisiac use erectile dysfunction or use by medical advice and use for treatment of erectile dysfunction.

### Quantitative results

Mean scores on 5-point Likert scale items ranged from 2.06 to 3.44. Table 2. Mean scores on the new survey items and the percentage of respondents agreeing with the culturally adapted perception of aphrodisiac use survey items indicate similarity between themes from the qualitative data and the subsequent quantitative data. Percentage of participants agreeing to the items ranged from 41.7 to 63.1% across all adapted perception of aphrodisiac use items. Item-total correlations for each adapted perception of aphrodisiac use item indicates the internal consistency of the newly developed items within their respective domains. All item-total correlations were statistically significant, except two reverse coded items. The significant Pearson’s correlation coefficient values ranged from − 0.33 to 0.70, with most items having moderate correlations. As expected, the negative correlations were for items worded to be in the opposite direction as compared to other items within the domain.

**Table 2:** Joint Display of Perception of Aphrodisiac use Questionnaire and corresponding University undergraduates focused Perception of Aphrodisiac Use items with sample qualitative phrase codes, corresponding themes, and quantitative mean of survey items

Perception of aphrodisiac use questionnaire survey Itemsb and domains	Themes from Qualitative Focus Groups	Sample phrase codes	Corresponding adapted survey Items	Item scores Mean ± SD (Percent agreement with item) (*n* = 919)	Item – total correlation Pearson’s Correlation Coefficient
**Without medical condition or medical advice**					
Aphrodisiac is used by sexually active adolescents/ youths without erectile dysfunction	Indications to use aphrodisiac	No indication for use except for prolonged sexual act attained through improvised products {R6_FGD_Male Undergrad_UI_ Hall 2} *There are indications for uses. Actually medically, they are some youths that really need it so it is medically indicated and recommended’* {*There are indications for uses. Actually medically, they are some youths that really need it so it is medically indicated and recommended’* {*KII* 3_Pharmacist_UI_IBNLGA}	Sexually active adolescents/youths without erectile dysfunction can use aphrodisiacs	2.20 ± 1.50 (56.9)	0.60**
Aphrodisiac is used by sexually active adults without erectile dysfunction	Indications to use aphrodisiac		Sexually active adults without erectile dysfunction can use	2.13 ± 1.44 (57.9)	0.58**
Aphrodisiac is used by sexually active elderly without erectile dysfunction	Indications to use aphrodisiac		Sexually active elderly without erectile dysfunction can use aphrodisiac is used by	2.35 ± 1.45 (49.0)	0.53**
**To treat erectile dysfunction**					
Aphrodisiac is used by people with erectile dysfunction	Indications to use aphrodisiac		People with erectile dysfunction can use aphrodisiac	3.25 ± 0.94 (54.2)	-0.21*
For erectile dysfunction	Indications for use of aphrodisiac substances		Aphrodisiac can be used for treatment of erectile dysfunction	3.40 ± 0.91 (62.5)	-0.33**
Aphrodisiac is used for medical reasons only e.g. erectile dysfunction	Indications for use of aphrodisiac substances		Aphrodisiac is used for medical reasons only e.g. erectile dysfunction	3.44 ± 0.87 (63.1)	-0.50**
Aphrodisiac should be used at all	Indications for use of aphrodisiac substances	Aphrodisiacs improve sexual performance through sustained and prolonged erection, vaginal tightening, sexual gratification and self-satisfaction {all participants}	Use of aphrodisiac should be completely avoided by youth	3.32 ± 0.98 (59.3)	-0.17*
**Recreational purpose** Aphrodisiac is used for recreational purpose for male	Benefits of using aphrodisiac		Male youth use aphrodisiac for recreational purpose	2.50 ± 1.51 (45.9)	0.58**
Aphrodisiac is used for recreational purpose for female	Benefits of using aphrodisiac		Female youth use aphrodisiac for recreational purpose	2.60 ± 1.49 (41.7)	0.57**
**Prolonged and improved sexual performance** Aphrodisiac is used for the gratification of sexual partner	Benefits of using aphrodisiac		Youth use aphrodisiac for gratification of sexual partner	2.16 ± 1.43 (56.7)	0.64**
Aphrodisiac is used to improve sexual performance	Benefits of using aphrodisiac		Aphrodisiacs use help improve sexual performance	2.06 ± 1.40 (60.0)	0.64**
Aphrodisiac is used to prolong/enhance erection	Benefits of using aphrodisiac		Aphrodisiac use prolong/ enhance erection	2.23 ± 1.45 (54.3)	0.64**
Aphrodisiac is used to boost self esteem	Benefits of using aphrodisiac		Aphrodisiac use boost self esteem	2.49 ± 1.1.49 (45.7)	0.67**
Aphrodisiac is used for self-satisfaction	Benefits of using aphrodisiac		Aphrodisiac use promote self-satisfaction	2.39 ± 1.48 (49.0)	0.70**
Aphrodisiac is used for prolonged sexual intercourse	Benefits of using aphrodisiac		Aphrodisiac use guarantee prolonged sexual intercourse	2.32 ± 1.1.50 (52.1)	0.65**

*Significant at 0.05 level (2-tailed) **Significant at 0.01 level (2-tailed)

### Psychometric analysis

**Fig. 1:**
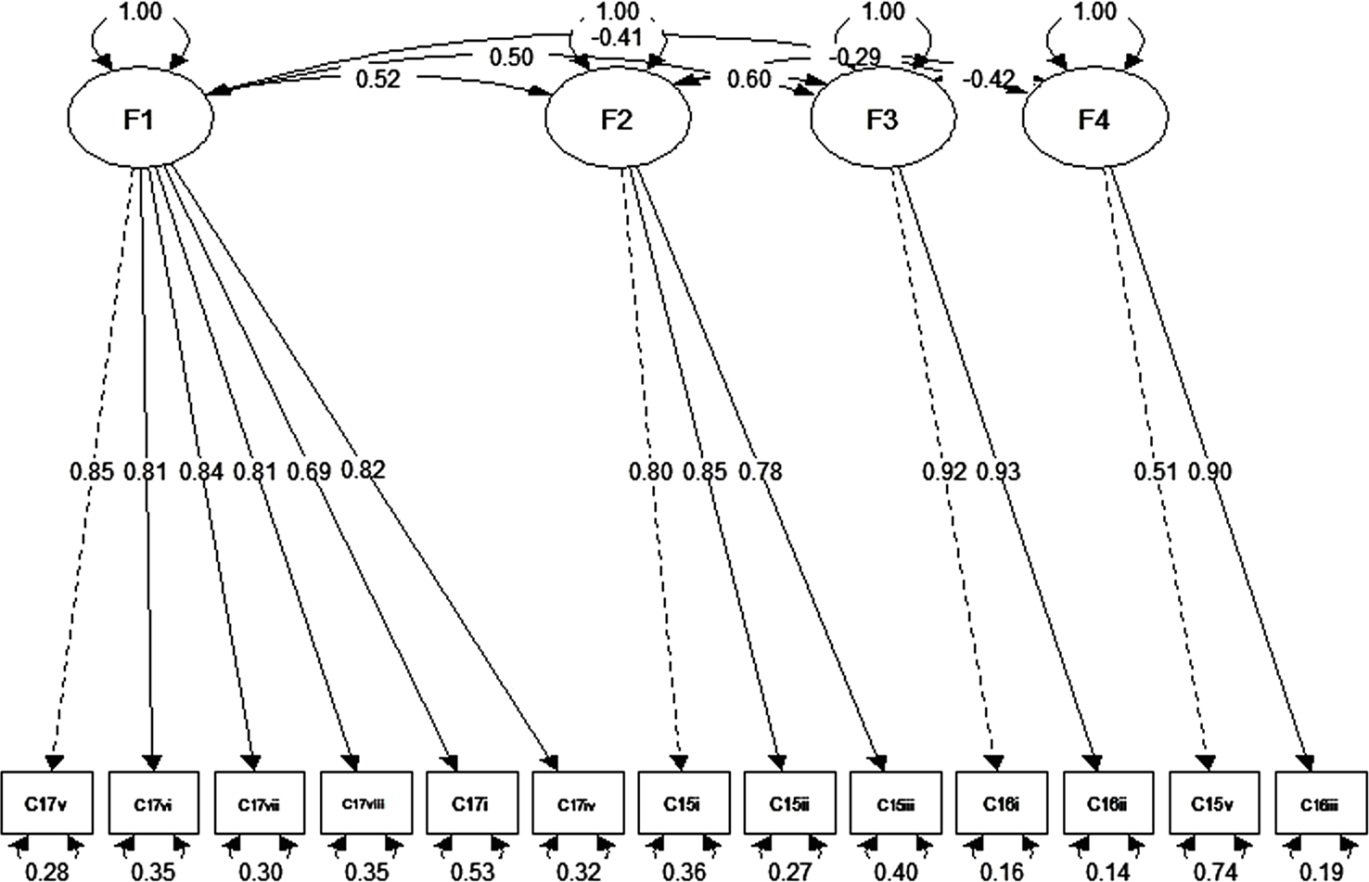
Polychoric correlation matrix plot of university student’s perception of aphrodisiac use. Items C15i, C15ii, and C15iii significantly correlated with one another having values that ranged between 0.66–0.73; items C16i and C16ii had a correlation value of 0.89; items C15iv and C16iii with a value of 0.51 moderately correlated with each and all items of C17 subtypes significantly correlated with one another in values that ranged from − 0.55 to 0.97

Initial polychoric correlation was analysed (Fig. 1) where items C15i, C15ii, and C15iii significantly correlated with one another having values that ranged between 0.66 and 0.73; items C16i and C16ii had a correlation value of 0.89; items C15iv and C16iii with a value of 0.51 moderately correlated with each and all items of C17 subtypes significantly correlated with one another in values that ranged from − 0.55 to 0.97.

Exploratory factor analysis was conducted using Maximum likelihood statistics for extracting factors. This yielded four factors from its minimum residual solution that rotated on its default oblimin transformation. These factors explain 66.31% of the variance in the original 15 variables.

The first batch of factor analysis on perception of use of aphrodisiac produced items of Factor 1 (C17v, C17vii, C17viii, C17vi, C17iv, C17ii, C17iii) which constitute “prolonged sexual performance”, questions C15ii, C15i, C15iii and C17i made up the scale “erectile dysfunction or by medical advice use”, questions C16i and C16ii made up the scale “recreational purpose” and questions C15iv and C16iii aggregated for “To treat erectile dysfunction”. The reliability test for the factors indicated excellent improvement when items C17i and C17iii were removed from the items of factors that connote aphrodisiac use for “erectile dysfunction or by medical advice and prolonged sexual performance” and “prolonged sexual performance”. Based on this outcome, a second round of factor analysis without the deleted items still suggested a four-factor solution with Kaiser-Meyer-Olkin (KMO) measure of 0.89. The factors and their respective Cronbach alpha values are Recreational purpose, α = 0.62; Aphrodisiac use without medical condition or medical advice, α = 0.84; To treat erectile dysfunction = 0.91 and Prolonged and improved sexual performance = 0.84. Table 3.

**Table 3:** Standardized loadings (pattern matrix) based on correlation matrix and reliability of factors generated from items of perception of use of aphrodisiac substances among undergraduate students of University of Ibadan

Reliability	Factor ID	Factor Description	Item	ML2	ML3	ML1	ML4	h2	u2	com
Alpha = 0.847 (3 items)	Factor 2	Without medical condition or medical advice	C15i. Aphrodisiacs should be used by sexually active adolescents/youths without erectile dysfunction	0.06	**0.75**	0.13	0.14	0.72	0.28	1.20
Alpha = 0.847 (3 items)	Factor 2	Without medical condition or medical advice	C15ii. Aphrodisiacs should be used by sexually active adults without erectile dysfunction	-0.01	**0.90**	-0.02	-0.03	0.79	0.21	1.00
Alpha = 0.847 (3 items)	Factor 2	Without medical condition or medical advice	C15iii. Aphrodisiacs should be used by sexually active elderly without erectile dysfunction	0.01	**0.78**	0.00	-0.13	0.67	0.33	1.10
Alpha = 0.627 (2 items)	Factor 3	To treat erectile dysfunction	C15iv. Aphrodisiacs should be used by people with erectile dysfunction	-0.06	-0.19	0.14	**0.61**	0.44	0.56	1.30
Alpha = 0.918 (2 items)	Factor 4	Recreational purpose	C16i. Aphrodisiacs should be used for recreational purpose for male	0.00	0.01	**0.98**	0.00	0.96	0.04	1.00
Alpha = 0.918 (2 items)	Factor 4	Recreational purpose	C16ii. Aphrodisiacs should be used for recreational purpose for female	0.02	0.04	**0.86**	-0.06	0.83	0.17	1.00
Alpha = 0.627 (2 items)	Factor 3	To treat erectile dysfunction	C16iii. Aphrodisiacs should be used for erectile dysfunction	-0.05	0.03	-0.21	**0.74**	0.68	0.32	1.20
Alpha = 0.915 (6 items)	Factor 1	Prolonged and improved sexual performance	C17ii. Aphrodisiacs should be used for gratification of sexual partner	**0.58**	0.23	0.09	0.11	0.57	0.43	1.40
Alpha = 0.915 (6 items)	Factor 1	Prolonged and improved sexual performance	C17iv. Aphrodisiacs should be used to improve sexual performance	**0.82**	0.00	-0.01	-0.10	0.72	0.28	1.00
Alpha = 0.915 (6 items)	Factor 1	Prolonged and improved sexual performance	C17v.Aphrodisiacs should be used to prolong/enhance erection	**0.88**	-0.01	-0.06	-0.11	0.79	0.21	1.00
Alpha = 0.915 (6 items)	Factor 1	Prolonged and improved sexual performance	C17vi. Aphrodisiacs should be used to boost self esteem	**0.84**	0.01	0.04	0.09	0.72	0.28	1.00
Alpha = 0.915 (6 items)	Factor 1	Prolonged and improved sexual performance	C17vii. Aphrodisiacs should be used for self-satisfaction	**0.87**	0.01	0.05	0.07	0.77	0.23	1.00
Alpha = 0.915 (6 items)	Factor 1	Prolonged and improved sexual performance	C17viii. Aphrodisiacs should be used for prolonged sexual intercourse	**0.86**	-0.05	0.00	-0.02	0.70	0.30	1.00

*ML1-ML4: Four generated factors from matrix of maximum likelihood factor analysis method

**h2: Communality scores–sum of the squared factor loadings for each question. Somewhat like an \(R^2\) value for each item

***u2: Uniqueness scores of communalities of items to factors (1-h2)

The bold values in the matrix pattern are significant correlations of items that loaded on each factors

**Fig. 2:**
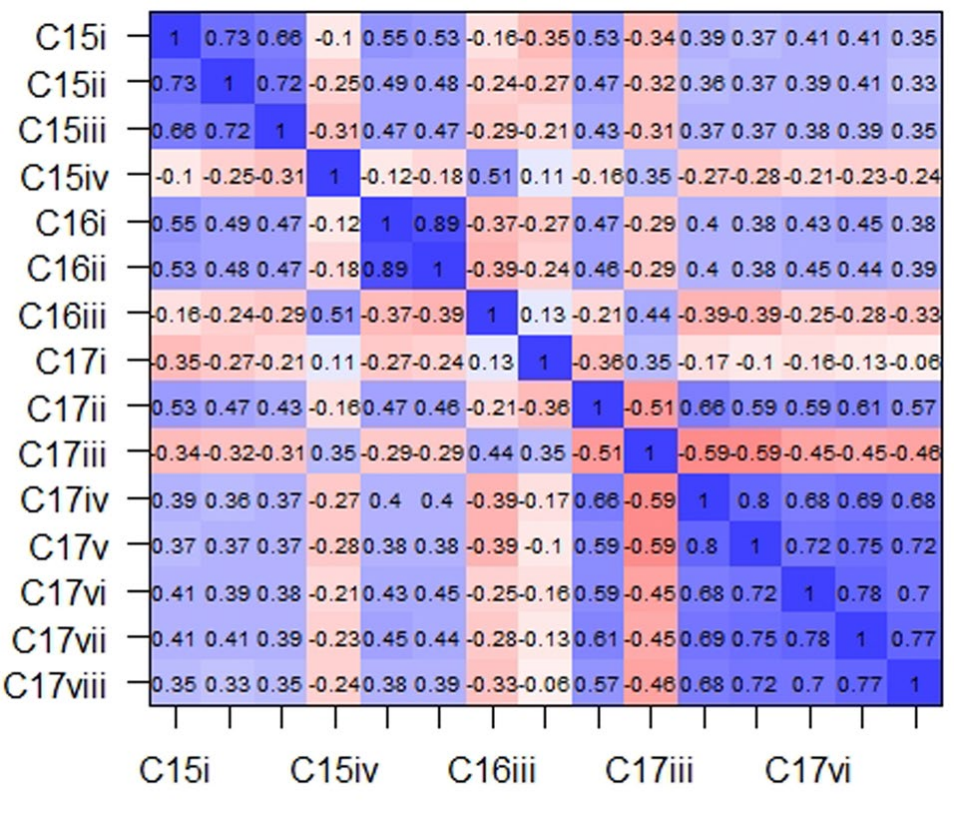
CFA path diagram of four factors of perception of aphrodisiac use of university students (F1 = prolonged sexual performance, F2 = erectile dysfunction or by medical advice use, F3 = recreational purpose, F4 = To treat erectile dysfunction) and their observed variables

A four-dimensional model derived from the exploratory factor analysis of thirteen items was subjected to CFA. All the CFA goodness of fit indices were within acceptable values except RMSEA that was just within marginal range (TLI = 0.931; CFI = 0.948; SRMR = 0.047; RMSEA = 0.083). Figure 2.

### Composite reliability

The composite reliability measure of each factor of perception of aphrodisiac use was strong (F1 = 0.90, F2 = 0.85, F4 = 0.92) except for factor 3 which indicated a moderate reliability (F3 = 0.63).

### Convergent and divergent validity of constructs

The inter item correlation of each of the constructs was significantly greater than 0.5 thus satisfying the convergence criteria.

The minimum correlation of each construct was greater than the correlations of respective inter-item correlations of adjoining constructs. By calculation, the total number of comparisons is 53 deduced from 6 × 6 + 3 × 3 + 2 × 2 + 2 × 2 and total violations are 0. According to Campbell and Fiske [29] the violation counts should be less than one-half the potential comparisons hence the discriminant holds valid.

Respondents’ perceptions of aphrodisiac use scores were converted to domain Z scores and assessed against respondents’ socio-demographic characteristics. Male students, those of Ibo ethnic group and having mothers with primary educational significantly all had poor perception of the domain of aphrodisiac use for prolonged sexual performance’s Z score. (-0.03 ± 0.02; -0.01 ± 1.00, *p* < 0.05; -0.01 ± 1.00). *p* < 0.05. Table 4.

In terms of age, those older than 20 years and male respondents had statistically significant weak differences in perception of use of aphrodisiac for condition of erectile dysfunction or by medical advice (-0.06 ± 1.01, *p* < 0.05; -0.11 ± 1.01, *p* < 0.05).

Poor Z mean score of perception of aphrodisiac use for treating erectile dysfunction statistically varied among the married, those of Ibo tribe, and educational status of respondents’ parents. Values decreased in a proportional manner to educational status of either parent. (-0.11 ± 1.20, *p* < 0.05; -0.40 ± 1.20, *p* < 0.05; -0.50 ± 1.14, *p* < 0.05, -1.0 ± 1.10). Table 4.

Significant statistical differences were also found between poor perception of recreational purpose use of aphrodisiac and the male students, Ibo ethnic group, mothers with primary education as well as in those earning above 20000 naira monthly. (-0.10 ± 1.03; -0.20 ± 1.10: -0.10 ± 1.00: -0.21 ± 1.02)

The physical and psychological symptoms of using aphrodisiacs were computed against the Z scores of factors derived from perception of its use. Table 4. Those with symptoms of sex addiction, priapism, performance, self-confidence and body weakness significantly varied with unsatisfactory Z mean measures of perception of aphrodisiac use for ‘without a medical condition or by medical advice’ construct. (-0.61 ± 0.66; -0.58 ± 0.81; -0.57 ± 0.86; -0.66 ± 0.69) *p* < 0.05. Weak standardized mean scores of perceptions of aphrodisiac use for recreational purpose statistically differed among respondents who had problems of sustained erection, body weakness, self-esteem and the perverts (-0.47 ± 1.01; -0.57 ± 0.80; -0.62 ± 0.92; -0.56 ± 0.92) *p* < 0.05.

**Table 4:** Comparative mean analysis of Z scores of perception of use of aphrodisiac’ domains with respondents’ socio-demographic characteristics and symptoms of aphrodisiac us

Characteristics		F1		F2		F3		F4	
Age group	*N* (%)	Mean ± SD	*p*-value	Mean ± SD	*p*-value	Mean ± SD	*p*-value	Mean ± SD	*p*-value
< OR = 20	385 (41.9)	0.04 ± 0.98	0.37	0.09 ± 0.98	**< 0.05**	0.03 ± 1.00	0.43	0.03 ± 1.00	0.38
> 20	534 (58.1)	-0.03 ± 0.02		-0.06 ± 1.01		-0.02 ± 1.00		-0.03 ± 1.00	
**Gender**									
Male	537 (58.40)	-0.11 ± 1.02	**< 0.05**	-0.11 ± 1.01	**< 0.05**	0.03 ± 1.00	0.22	-0.10 ± 1.03	**< 0.05**
Female	382 (41.6)	0.20 ± 1.00		0.12 ± 1.10		-0.4 ± 1.10		0.11 ± 1.04	
**Marital status**									
Single	885 (96.3)	-0.01 ± 1.00	0.38	0.00 ± 1.00	0.8	0.02 ± 1.00	**< 0.05**	-0.00 ± 1.00	0.81
Married	34 (3.7)	0.20 ± 1.10		-0.04 ± 1.20		-0.11 ± 1.20		0.04 ± 1.01	
**Ethnicity**									
Yoruba	676 (73.6)	-0.01 ± 1.00	**< 0.05**	-0.01 ± 1.00	0.06	0.10 ± 1.04	**< 0.05**	-0.02 ± 1.00	**< 0.05**
Ibo	72 (7.8)	-0.20 ± 1.20		-0.20 ± 1.00		-0.40 ± 1.20		-0.20 ± 1.10	
Hausa	117 (12.7)	-0.01 ± 1.00		0.10 ± 1.14		-0.10 ± 1.10		0.10 ± 1.10	
Others	54 (5.90)	0.40 ± 1.00		0.30 ± 1.03		-0.40 ± 1.10		0.40 ± 1.00	
**Father’s educational level**									
No formal education	100 (11.5)	-0.03 ± 1.10	0.3	-0.1 ± 1.10	0.57	-0.40 ± 1.10	**< 0.05**	0.20 ± 1.03	0.57
Primary	64 (7.4)	0.24 ± 1.10		-0.10 ± 1.00		-0.50 ± 1.14		0.11 ± 1.00	
Secondary	156 (17.9)	0.10 ± 1.04		0.10 ± 1.00		0.02 ± 1.00		0.03 ± 1.02	
Tertiary	550 (63.2)	0.01 ± 1.00		0.10 ± 1.00		0.10 ± 1.00		0.01 ± 1.0	
**Mother’s educational level**									
No formal education	96 (11.2)	0.30 ± 1.12	**< 0.05**	0.10 ± 1.10	0.08	-1.0 ± 1.10	**< 0.05**	0.32 ± 1.02	**< 0.05**
Primary	90 (10.5)	-0.20 ± 1.02		-0.22 ± 1.00		-0.24 ± 1.10		-0.10 ± 1.00	
Secondary	175 (20.4)	0.10 ± 1.04		0.02 ± 1.00		-0.01 ± 1.00		0.04 ± 1.01	
Tertiary	498 (58.0)	-0.01 ± 1.00		0.10 ± 1.00		0.14 ± 1.00		0.01 ± 1.00	
**Average monthly income (Naira)**									
≤ 20,000	380 (75.8)	-0.10 ± 1.00	0.25	-0.01 ± 1.00	0.32	0.05 ± 1.00	0.43	0.01 ± 1.00	**< 0.05**
> 20,000	121 (24.2)	-0.20 ± 1.00		-0.11 ± 1.04		0.13 ± 1.05		-0.21 ± 1.02	
**Sex Addiction**									
Yes	69 (57.0)	-0.35 ± 0.89	0.34	-0.61 ± 0.66	**< 0.05**	-0.11 ± 0.86	0.32	-0.33 ± 1.00	0.37
No	52 (43.0)	-0.19 ± 0.89		-0.04 ± 1.09		0.07 ± 1.07		-0.17 ± 0.95	
**Priapism**									
Yes	59 (48.8)	-0.58 ± 0.81	1.4	-0.58 ± 0.81	**< 0.05**	-0.06 ± 0.95	0.76	-0.47 ± 1.01	**< 0.05**
No	62 (51.2)	-0.17 ± 0.96		-0.17 ± 0.96		0.01 ± 0.96		-0.06 ± 0.89	
**Loss of self-confidence**									
Yes	56 (46.3)	-0.37 ± 0.94	0.3	-0.57 ± 0.86	**< 0.05**	-0.01 ± 0.91	0.62	-0.13 ± 0.90	0.13
No	65 (53.7)	-0.20 ± 0.84		-0.19 ± 0.92		-0.13 ± 0.90		-1.13 ± 0.90	
**Aphrodisiac dependent**									
Yes	52 (43.0)	-0.35 ± 0.98	0.45	-0.53 ± 0.96	0.09	-0.01 ± 1.01	0.68	-0.46 ± 0.98	0.52
No	69 (57.0)	-0.23 ± 0.81		-0.25 ± 0.86		-0.07 ± 0.92		-0.11 ± 0.93	
**Causes you weakness**									
Yes	45 (37.2)	-0.26 ± 0.94	0.89	-0.66 ± 0.69	**< 0.05**	-0.53 ± 0.93	0.88	-0.57 ± 0.80	**< 0.05**
No	76 (62.8)	-0.29 ± 0.86		0.20 ± 0.1.00		-0.03 ± 0.98		-0.08 ± 1.02	
**Loss of self-esteem**									
Yes	43 (35.5)	-0.33 ± 0.97	0.64	-0.54 ± 0.93	0.11	1.49 ± 0.92	0.13	-0.62 ± 0.92	**< 0.05**
No	78 (64.5)	-0.25 ± 0.85		-0.27 ± 0.89		-0.12 ± 0.97		-0.07 ± 0.95	
**Perversion**									
Yes	50 (41.3)	-0.36 ± 0.97	0.5	-0.46 ± 0.92	0.51	-0.09 ± 1.00	0.49	-0.56 ± 0.92	**< 0.05**
No	71 (58.7)	-0.25 ± 0.83		-0.35 ± 0.86		-0.06 ± 0.96		-0.10 ± 95	

The bold values are p-values of significant differences between the independent variables and the factors mean scores

## Discussion

### Quantitative

The average item scores and significant item-total correlations were important indicators of the initial validity and reliability of the culturally adapted survey items in this population. Most items had average scores ranging from 2.10 to 3.32, (with the extreme means being 2.00–3.44) an indication that participants in general found the items relevant to their perception of aphrodisiac use. There are no extreme values among the items an indicator of item quality when the goal of the assessment is normative.

Two items (people with erectile dysfunction can use aphrodisiac, use of aphrodisiac should be completely avoided by youth) indicated poor item-total correlation with the domain of indications for use of aphrodisiac. These items were retained for further evaluation in construct validity to determine if they are internally valid with the construct or to be deleted from the survey items if they are not.

### Qualitative

The initial qualitative phase was essential to explore perception of aphrodisiac use that may be peculiar to undergraduate youths in Nigeria university. Themes on perception of aphrodisiac use such as indications for using aphrodisiac and benefits of using aphrodisiac and as well as themes on medical and psychological impacts of using aphrodisiac were all informed by environmental awareness and experiences common to students of tertiary institutions. It was important to explore the perception of using aphrodisiac because of the connectedness they have with health and psychological impact on young users [14, 17]. By addressing these unique perceptions among students of higher learning, it would allow for creation of evidence based interventions for protective and responsible behaviours in sexual activities.

Most research findings done in West Africa indicated that the major reason for using aphrodisiacs is the excitatory psychological benefits they render to heightening of sexual pleasures and its compensating benefits and challenges that re-trigger the cycle of use [5–10]. The other reason for aphrodisiac use is the medical indication of erectile dysfunction which could be caused by biological and behavioural underlining factors [12, 13, 19]. Participants in this study discussed their views about indication for use of aphrodisiacs by undergraduate youths. They obviously do not reckon with the therapeutic use of except for the diverse opinions of KII participants that ranged between belief that there are indications for the use among youths and no indication for aphrodisiac use by the specified populations. The theme on benefits of aphrodisiac use, however, received huge perceptive agreements among all categories of discussants. The essence of sexual engagements which is not subjected to any particular age groups except among juveniles could explain the strong perception and tendency to usage of aphrodisiacs among discussants which may have been derived from testimonies of peers or personal experimentation [5–10]. These major themes shaping perception of aphrodisiac use are accounted for in the adapted survey instrument.

The remarkable description of knowledge of psychological and medical impacts of aphrodisiac use by majority of participants portray that youths perceive immediate or latter life dangers in uncontrolled and habitual use of the substances [14, 17]. This offers a window of opportunity to design public health interventions that will promote health sexual choices and lifestyle for the youth.

### Integration

Integration of qualitative and quantitative data occurred at two phases, first when using the building approach to create newly adapted (university undergraduates focused) items and then when using the merging approach to report the results through the joint display. The cultural adaptation evolved from qualitative themes for composition of quantitative survey items, as well as the creation of new items. Creating uniquely adapted items based on in-depth qualitative data resulted in enhanced content validity of the items. Matching the themes to the theoretical domains of perception of aphrodisiac use led to an approach that dovetailed to development and structuring of final survey items.

Although there is no guarantee that research structure of this study for the population of interest will be the same if applied to general population. However, the construct validity evaluated through structural equation modelling of the domains’ items by this study could suffice for the measurement of the perception of aphrodisiac use among the population of interest in any African university. It could be used at such locations after conducting prior pretest for psychometric validity for further fine-tuning of the instrument.

### Psychometric analysis

The main objective of the psychometric analysis was to develop and validate perception of aphrodisiac use from university undergraduates from the original survey instrument. A four-factor structure for 13 out of the 15 items was evident, based on a principal components exploratory factor analysis with an oblimin rotation of the scale’s polychoric correlation matrix.

The fitted model for perception of aphrodisiac use among university undergraduates proposed four-factor structure, involving prolonged and improved sexual performance (6 items; 0.915), without medical condition or medical advice (3 items; 0.847), to treat erectile dysfunction (2 items; 0.627), Recreational purpose (2 items; 0.918) factors. This indicates good internal consistency except a questionable factor 3. However, each of the factors could probably be made robust by further refining of items for the purpose of clarity to the target audience. The two items under factor 3 implied the same meaning indicating need to delete one of them and to substitute it with one or more combinations of new items that could align to and elicit same construct.

The four-factor and 13 items scale that were extracted through EFA was validated by CFA to establish a relationship between the exogenous variables (items of perception of aphrodisiac use) and the endogenous latent constructs vis-a-viz acceptable goodness of fit measures. The first and only CFA done as suggested by the EFA produced the fit to the model. Perception in psychology is one of the key attributes of attitude which forms human habit. All components that made up attitude were transactions with one’s physical and social surroundings and that the direction of influence flowed either direction ways—our attitudes are influenced by the social world and our social world is influenced by our attitudes. With respect to the outcome of the psychometrics validation of this study, a theoretical framework has been established as different items on perception of realities of aphrodisiacs aggregated into four constructs that could influence its habitual use. Thus, future studies can examine these domains with respect to use of aphrodisiac substances particularly among youths in institutions of higher learning. This study was also conducted to also assess the validity of the perception of aphrodisiac use among tertiary education students by their socio-demographic characteristics and consequences of its use. This is to determine factors that could influence perception of aphrodisiac use among young adults when the validated instrument is applied. The observed differences in mean scores of the perception of aphrodisiac use among the subjects further underscore the need to use the domains described for comparison of each subgroup of independent variables. With regards to socio-demographic characteristics of respondents, being a male and of Ibo ethnic background presented poorly in all constructs of perception of aphrodisiac use except in respective perception of “aphrodisiac use for treating erectile dysfunction and use of aphrodisiac for condition of erectile dysfunction or by medical advice”. The different findings with the male sex transcend cultural and socioeconomic status since several qualitative and quantitative studies focusing on factors predisposing perception and use of aphrodisiacs established this natural phenomenon [11, 24]. Universally, men have the propensity for aiming to achieve sexual satisfaction characterized by prolonged pleasure to prove their phallic capabilities to themselves and to the opposite sex.

The marked variations between the married, young adults and those from non to early educated parents with poor perceptions of use of aphrodisiac for condition of erectile dysfunction or by medical advice and treating erectile dysfunction are realistic associations. For the married, undesirable sexual experience is one of the underlying problems causing marital crisis that could prompt preference for aphrodisiac substances to ameliorate the condition [25, 26]. Studies by Oniye et al. (2016) [25] and Sanni [26] have investigated the use and pattern of aphrodisiacs by couples to correct sexual dysfunction. The fact that individuals above 20years significantly had poor perception of aphrodisiac use have been reported in previous literature [9, 27]. The demand for and dependency on aphrodisiac for sexual pleasure or medical reason is higher among this group [9]. The indication for mothers’ lowered educational status and the identified domains of aphrodisiac perception among the population of interest could be considered in future assessment of the scale. studies have not been conducted to evaluate this area, it would be interesting to investigate how parental higher level of education influences young adults’ perception of aphrodisiac use and its medical implications. The significant variation of the higher monthly earning of the students with construct of perception of use of aphrodisiac for recreational purposes should be explored in future use of the scale. This could help in understanding how financial wherewithal could shift the perception of aphrodisiac use among undergraduate students.

The scale was able to differentiate between subgroups of physical and psychological consequences of using aphro­disiac, suggesting a good construct validity of this instrument designed to measure the multi-domain perception of aphrodisiac use. Those who have sex addiction, priapism, lack of sexual performance self-confidence, body weakness, loss of self-esteem and perversion from using aphrodisiacs significantly had worse scores on perception of aphrodisiac use without a medical condition or by medical advice and for recreational purposes.

Several studies have linked abuse of aphrodisiac substances to venereal diseases, psychological health conditions and simple to life threatening non-communicable diseases such as heart failure and kidney problems ] [17, 28, 30–34]. Manortey et al. [7] reported that despite health regulatory bodies’ issuance of public awareness on concerns for abuse of unregistered sex enhancing products, purchase remains high amidst menacing side effects. This report corroborated our study’s findings and they all implied that having health problems associated with use of aphrodisiac and the knowledge thereof are not important to having right perception to use of aphrodisiac. Based on this premise, it would be worthwhile, for future use of this scale to assess the significance of health outcomes of aphrodisiac users against the underlying perception to its use.

### Limitations

A number of limitations of the study can be spotlighted. The findings of this study cannot yet be fully generalized due to the wording and interpretation of specific items that aggregated to some factors and the restriction to student population of just one university. Nevertheless, research efforts should be extended to detail and thorough exercise on the scales’ items development and testing in a variety of tertiary educational institutions in order to enhance its robustness and flexibility for generalizability purposes. Another limitation of the research is the restricted number of items that statistically constitute some factors, which may cause availability of limited or no options for researchers, should removal of items be required.

## Conclusion

This study used a mixed methods design and construct validity analysis to develop and validate a survey instrument on perception of aphrodisiac use among students of a Nigerian tertiary institution. The mixed methods validation of the survey for measuring perception of aphrodisiac use among the undergraduates led to statistically significant integration of quantitative domain items with thematic domains of qualitative analysis. The construct validity indicated that all the domains that emerged from the analysis statistically reflect the stimuli, cognitive, translation, behaviour and performance components of perception to aphrodisiac use. The four-factor generated model showed a good fit, indicating that sexual elongation pleasure, use of aphrodisiac substances without erectile dysfunction, treatment of erectile dysfunction and recreational purpose are adequate components to measure the perception of aphrodisiac use among university undergraduates. The resulted 13 items showed valid factors loadings and high values of internal consistency and reliability reinforced through CFA procedures. There are evidences of statistically significant differences between groups of certain socio-demographic characteristics and consequences of aphrodisiac with domains of perception of aphrodisiac use.

Equally, all the items of the four constructs from the reliable construct validity outcomes also aligned with the integrated mixed method results.

## Data Availability

The datasets used and/or analysed during the current study available from the corresponding author on reasonable request.
